# Long-Term Consumption of 10 Food Groups and Cardiovascular Mortality: A Systematic Review and Dose Response Meta-Analysis of Prospective Cohort Studies

**DOI:** 10.1016/j.advnut.2022.10.010

**Published:** 2022-12-22

**Authors:** Buna Bhandari, Zhixin Liu, Sophia Lin, Rona Macniven, Blessing Akombi-Inyang, John Hall, Xiaoqi Feng, Aletta E. Schutte, Xiaoyue Xu

**Affiliations:** 1 School of Population Health, University of New South Wales, Sydney, Australia; 2 Central Department of Public Health, Tribhuvan University Institute of Medicine, Kathmandu, Nepal; 3 Department of Global Health and Population, Harvard T.H. Chan School of Public Health, Boston, USA; 4 Stats Central, University of New South Wales, Sydney, Australia; 5 Ministry of Health, New South Wales, Sydney, Australia; 6 The George Institute for Global Health, Sydney, Australia

**Keywords:** grains, vegetables, fruits, meat, legumes, nuts, cardiovascular mortality, diet, nutrition

## Abstract

A large body of evidence exists on diet and cardiovascular mortality, but limited studies have investigated the long-term intake of food groups, which may have cumulative effects on cardiovascular health in the long term. This review therefore evaluated the relationship between the long-term consumption of 10 food groups and cardiovascular mortality. We conducted a systematic search in Medline, Embase, Scopus, CINAHL, and Web of Science till January 2022. Of the 5318 studies initially identified, 22 studies with a total of 70,273 participants with cardiovascular mortality were included. Summary HRs and 95% CIs were estimated using a random effects model. We found that a long-term high intake of whole grains (HR: 0.87; 95% CI: 0.80, 0.95; *P* = 0.001), fruits and vegetables (HR: 0.72; 95% CI: 0.61, 0.85; *P* < 0.0001), and nuts (HR: 0.73; 95% CI: 0.66, 0.81; *P* < 0.00001) significantly reduced cardiovascular mortality. Each 10-gram increase in whole grain consumption per day was associated with a 4% reduction in the risk of cardiovascular mortality, whereas each 10-gram increase in red/processed meat consumption per day was associated with a 1.8% increase in the risk of cardiovascular mortality. Compared with the lowest intake category, red/processed meat consumption in the highest category was associated with an increased risk of cardiovascular mortality (HR: 1.23; 95% CI: 1.09, 1.39; *P* = 0.006). High intake of dairy products (HR: 1.11; 95% CI: 0.92, 1.34; *P* = 0.28), and legumes (HR: 0.86; 95% CI: 0.53, 1.38; *P* = 0.53) were not associated with cardiovascular mortality. However, in the dose-response analysis, each 10-gram increase in legume intake per week was associated with a 0.5% reduction in cardiovascular mortality. We conclude that the long-term high intake of whole grains, vegetables, fruits, nuts, and a low intake of red/processed meat are associated with reduced cardiovascular mortality. More data on the long-term effects of legumes on cardiovascular mortality are encouraged.

This study was registered at PROSPERO as CRD42020214679.

**Statement of Significance**

This systematic review and meta-analysis with a dose-response analysis provides comprehensive information on long-term consumption of 10 food groups and cardiovascular mortality. We conclude that the long-term high intake of whole grains, vegetables, fruits, and nuts and low intake of red and processed meat are associated with reduced cardiovascular mortality.

## Introduction

CVD is the leading cause of death and disability globally [1]. It accounted for 18.6 million preventable deaths in 2019 [2], which is one-third (32%) of the total number of global deaths [1]. The link between some risk factors and CVD have been well established, with a poor diet identified as a key risk factor [3]. According to the Global Burden of Disease Study 2019, dietary risk was the second leading cause of cardiovascular mortality, responsible for >7.94 million cardiovascular deaths worldwide [2].

Some important aspects of dietary risk have not been thoroughly investigated in previous studies. First, limited studies have reviewed long-term dietary consumption (>one time point dietary measurements) in relation to cardiovascular mortality, with most studies commonly linking one time point dietary consumption measurement to mortality. However, using one data point cannot determine long-term dietary habits and does not permit distinction between cause and effect [4]. Second, previous reviews have often analyzed the effect of dietary patterns, such as the Mediterranean diet, DASH diet, or individual food items in relation to cardiovascular mortality risk [5,6]. Although many national dietary guidelines used for population health promotion activities are based on food groups rather than food patterns [7,8], there are limited collective evidence that have synthesized the risk of different food groups and cardiovascular mortality, particularly focusing on the effect of long-term consumption of different food groups and cardiovascular mortality. From a public health perspective, diet-disease relationships can be better understood through the study of specific food groups [9]. Third, although it is known that there are sex-specific food choice preferences for energy and nutrient intake, limited studies have reviewed sex-differences of dietary consumption concerning cardiovascular mortality [10].

Therefore, we conducted a systematic review and meta-analysis with a dose-response analysis with robust inclusion criteria with restrictions to only analyze cohort studies that had repeated measures of dietary intake (≥two data collection points) throughout the study period. We analyzed the relationship between the long-term consumption (>5 yr) of the main food groups defined a priori as whole grains, vegetables, fruits, nuts, legumes, eggs, poultry, dairy products, fish/seafood, red/processed meat, and cardiovascular mortality, stratified by sex (where possible).

## Methods

This systematic review and meta-analysis adhered to the PRISMA 2020 guidelines [11] (Supplemental File 1). This review is registered in the PROSPERO [CRD42020214679].

### Search strategy

The search was conducted using the electronic databases MEDLINE, EMBASE, CINAHL, Scopus, and Web of Science. The details of the search terms used are provided in Supplemental Methods. In addition, references from the retrieved articles, including systematic reviews and meta-analyses, were manually checked for eligibility and inclusion. All searched studies were exported to Covidence software. Each abstract and title screening were performed by two of four independent reviewers (BB, XX, SL, and RM) and full texts were reviewed by BB and XX. Any disagreements were resolved by consensus after discussion.

### Study selection

Studies were included in the systematic review and meta-analysis if they 1) were prospective cohort studies; 2) were peer-reviewed and where the full text was available; 3) provided information about the association of food groups, including whole grains, vegetables, fruits, nuts, legumes, eggs, poultry, dairy products, fish/seafood, red/processed meat. These 10 food groups are the focus because they form the basis of most diet quality indexes or scores, and have been commonly reported in guidelines [7] and previous studies [9]; 4) included participants aged ≥18 yr; 5) considered cardiovascular mortality as an outcome; 6) measured the exposure of dietary consumption at >one time point; 7) written in English; and 8) were published between January 2000 and January 2022. We excluded studies that measured the dietary consumption at only one timepoint or reported only all-cause mortality without specifying cardiovascular mortality.

### Data extraction

During the screening process, duplicate records were removed, followed by the screening of records based on titles and abstracts. In the final screening phase, the full texts of articles were obtained, and the articles meeting the inclusion criteria were retained. Two independent reviewers (BB and XX) extracted the following information from the included studies: first author name, year of publication, country where study conducted, cohort study name, sample size, number of subjects, age at entry, sex, study duration (follow-up in years), outcomes, outcome assessment, assessment of food group, quantity of food consumed per day per individual, risk estimate [most adjusted measures; HRs, RRs, ORs with their corresponding 95% CIs], and variables that were adjusted for.

If there were several risk estimates provided, HRs/RRs/ORs in the multivariable adjusted model were extracted for the meta-analysis. The most common adjusted factors were age, sex, current smoking status, BMI, alcohol intake, and physical activity. If there were separate findings or risk estimates for male and female participants presented in a study, we extracted these to include separately in the meta-analysis. The details of extracted articles are shown in the Supplemental Tables 1–7.

### Quality assessment

We used the Newcastle-Ottawa quality Assessment Scale to assess studies’ quality given it commonly used to evaluate the quality of cohort studies [12]. We assessed study quality based on: how the studies ascertained exposure, how they assessed outcomes, whether follow-up time to mortality was adequate (>10 yr in most of the included studies), and whether they included an unadjusted model and made any other relevant adjustments (e.g., age, sex, education, BMI, smoking, and physical activity). A maximum of 9 points was given based on three scoring domains, including cohort selection (4 points), the comparability of the cohort design and analysis (2 points), and the adequacy of outcome measures (3 points). Detailed scoring criteria have been explained in the tool [12]. A total score of >6 points was considered good quality. The detailed scores have been calculated and shown in Supplemental Tables 1–7. Two reviewers (BB and XX) assessed the risk of bias independently. Disagreements in score allocations were resolved by discussion and consensus.

### Statistical analyses

A random-effect model meta-analysis was performed to pool combined HRs/RRs/ORs of the association between each food group intake and cardiovascular mortality. The highest category of food intake was compared with the lowest category of food intake (reference group) using the generic inverse-variance method. The actual amount of food intake was also converted into grams in the dose-response analysis as described below. Statistical heterogeneity between the cohort was quantified with the use of the *I*^2^ statistic; and *I*^2^ > 50% indicated evidence of considerable heterogeneity. A funnel plot was used to explore the potential small-study effects, such as publication bias. Due to the small number of studies (<10) included in each food group meta-analysis, an Egger test was not performed, as recommended by the Cochrane Handbook [13].

For the dose-response analysis, when food consumption was reported by the intake range, the midpoint of the range was used. If the upper boundary of the highest category was not provided, the width of the category was assumed to be the same as the adjacent category. The two-stage random-effect model was used to examine the linear and nonlinear dose-response relationship between food consumption and cardiovascular mortality. The Generalized least squares regression proposed by Greenland and Longnecker [14] and Orsini et al. [15] was initially used to estimate the trend of effect measure (HR); nonlinearity was then examined based on restricted cubic splines with three knots (25, 50, and 75th percentiles). The dose-response analysis could only be conducted on red/processed meat, whole grain, legumes, and dairy product groups. Nuts, eggs, and fruits and vegetables groups did not have sufficient studies for the dose-response analysis.

The meta-analysis was performed with the use of Review Manager software (Revman, version 5.4; The Nordic Cochrane Centre, The Cochrane Collaboration) and dose-response analysis was performed in R software.

## Results

**FIGURE 1:**
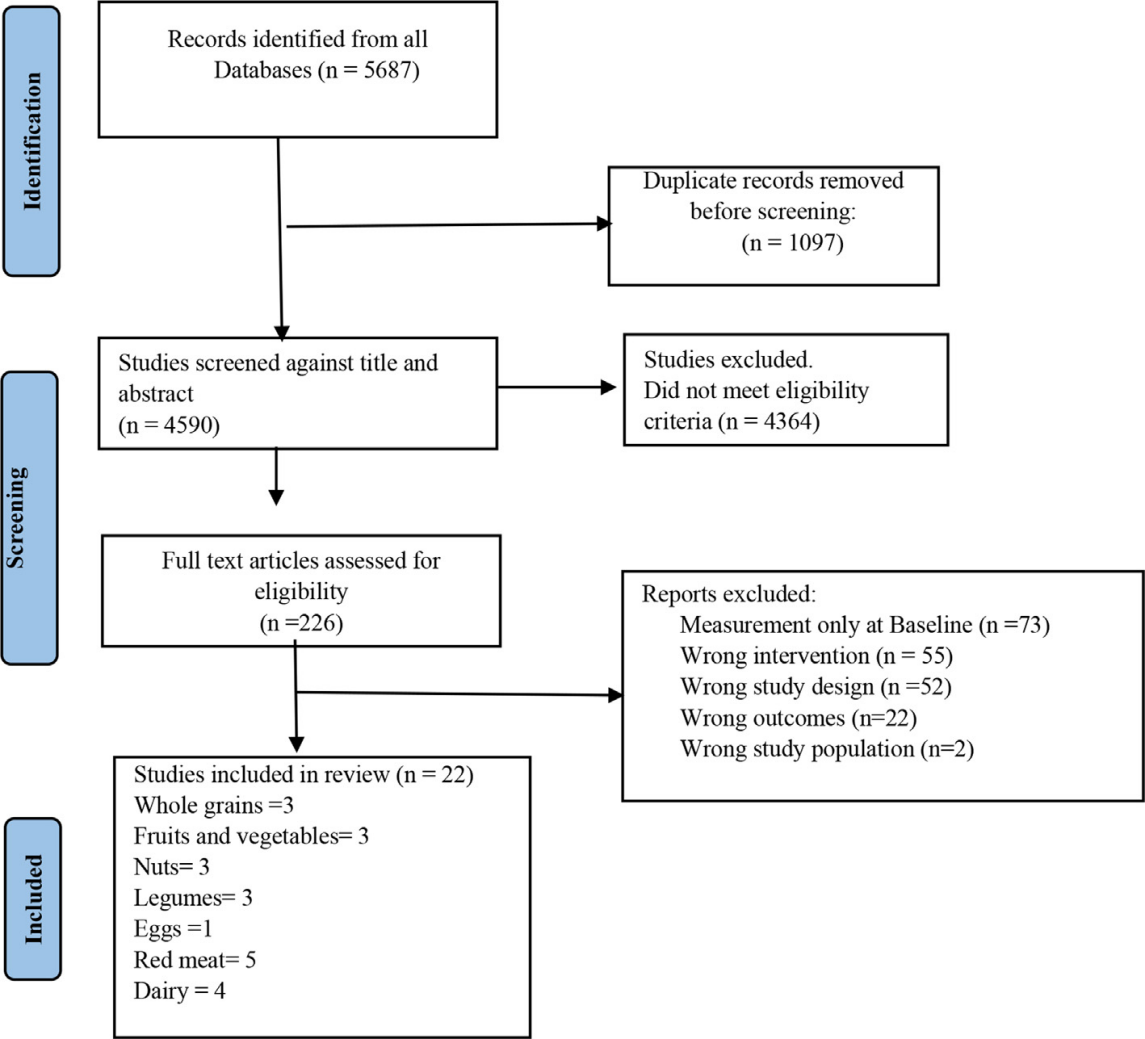
PRISMA flowchart of study selection

Of the 5687 records that were identified from the literature search, 226 full-text articles were assessed in detail because they reported cardiovascular mortality and different food groups in the title or abstract (Figure 1). After a full-text review, a total of 22 studies were included for data extraction based on the review eligibility criteria. Among the included studies, three studies reported consumption of grains [[16], [17], [18]] (Supplemental Table 1), six studies for red/processed meat and eggs [[19], [20], [21], [22], [23], [24]] (five red meat, one egg) (Supplemental Table 2), four dairy product studies [[25], [26], [27], [28]] (Supplemental Table 3), three nut consumption studies [[29], [30], [31]] (Supplemental Table 4), three legume studies [[32], [33], [34]] (Supplemental Table 5), two fruit, [35,36], and one vegetable study [37] (Supplemental Table 6). There were no studies for the food groups of fish/seafood and poultry based on our inclusion criteria of repeated measurements.

### Characteristics of included studies

The characteristics of the included studies are shown in Supplemental Tables 1–6. Ten studies were conducted in the United States [17,18,[20], [21], [22],^26^,[28], [29], [30], [31]], three in Australia [27,36,37], three in Japan [16,24,32], two in Sweden [19,25], one in United Kingdom [23], one in China [35], one in Spain [33], and one in Iran [34]. The length of follow-up ranged from 6 to 34 yr. Eighteen studies included both males and females (of these, only 38% reported sex-specific results), whereas three had only females and one had only male study participants. Regarding the dietary data collection methods, 21 of 22 studies used a self-administered food frequency questionnaire, and 1 study [35] used an interviewer-administered questionnaire. All included studies were of high quality having a score of 6 or above based on the Newcastle-Ottawa risk of bias assessment studies. Almost all the studies [21] reported that the analysis was adjusted for potential confounders (Supplemental Tables 1–6).

### Whole grains

**FIGURE 2:**
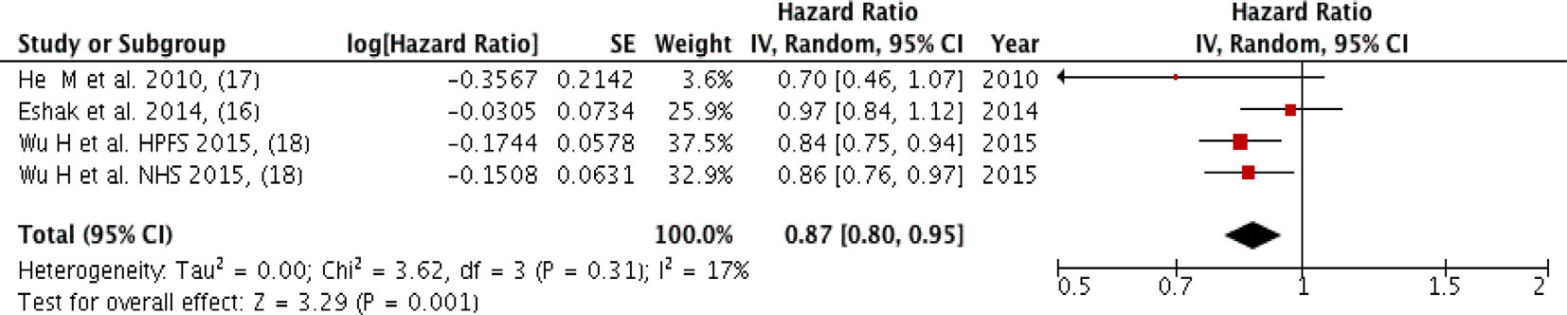
Forest plot showing multivariate adjusted HR with 95% CIs for the highest versus the lowest whole grains consumption and cardiovascular mortality in adults. 95% CI calculated from random effect models; IV, Inverse variation; pooled estimates of >1 favor higher consumption and of <1 favor lower consumption; NHS, Nurses’ Health Study; HPFS, Health Professional Health study.

Three studies (four groups) with a total of 9610 cardiovascular mortality cases were included in the meta-analysis, comparing the highest intake to the lowest intake. Figure 2 shows an inverse association between cardiovascular mortality and whole grain intake that was observed while comparing the extreme categories (Quintile 5 versus Quantile 1) with low heterogeneity among studies (Pooled HR: 0.87; 95% CI: 0.80, 0.95; *P* = 0.001; I^2^ = 17%, *P*-heterogeneity = 0.31). However, one of the studies only included females with type 2 diabetes mellitus [17], (Supplemental Table 1). There was no severe asymmetry observed from the visual inspection of the funnel plot (Supplemental Figure 1).

**FIGURE 3:**
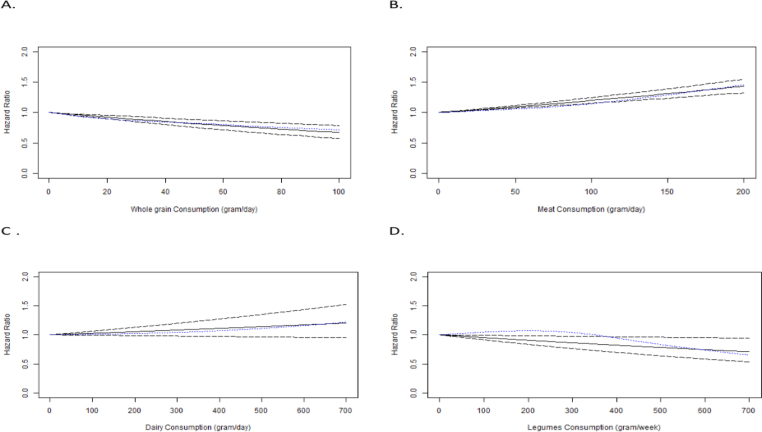
Linear dose–response relation between daily intakes of (A) whole grains, (B) red/processed meat, (C) dairy products, and (D) legumes as well as risk of cardiovascular mortality in adults**.** Solid lines represent linear trend, dashes represent CIs, and dotted blue line represent cubic spline.

The dose-response analysis of the three included studies (four groups) showed that for each 10-gram per day increase in whole grain consumption, there is a 4% reduction in the risk of cardiovascular death (HR: 0.96; 95% CI: 0.95, 0.98) (Figure 3A) with a significant decrease in the risk of cardiovascular death (*P* < 0.0001) with higher grain intake. The nonlinear trend was nonsignificant (*P* = 0.23) (Supplemental Table 7).

### Red and processed meat

**FIGURE 4:**
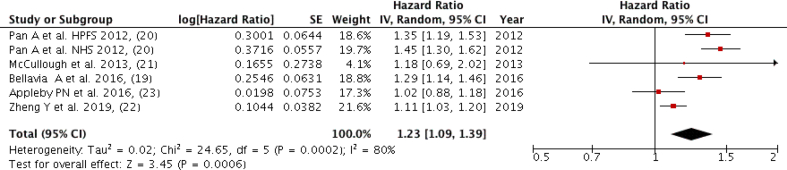
Forest plot showing multivariate adjusted HR with 95% CIs for the highest vs. the lowest red/processed meat consumption and cardiovascular mortality in adults. 95% CI calculated from random effect models; IV, Inverse variation; pooled estimates of >1 favor higher consumption and of <1 favor lower consumption; NHS, Nurses’ Health Study; HPFS, Health Professional Health study.

Five studies (six groups) with a total of 15,651 cardiovascular mortality cases were included in the meta-analysis, comparing the highest intake to the lowest intake (Figure 4). A positive association was found between red and processed meat intake and cardiovascular mortality while comparing the highest and lowest categories (Quintile 5 versus Quantile 1) with a high heterogeneity among the studies (Pooled HR: 1.23; 95% CI: 1.09, 1.39; *P* = 0.0006; I^2^ = 80%, *P*-heterogeneity = 0.0002) (Figure 4). There was no severe asymmetry observed from the visual inspection of the funnel plot (Supplemental Figure 2).

The dose-response analysis of the four included studies (five groups) [excluding the Zheng et al. [22] study due to not having category-wise HR required for dose-response] showed for each 10-gram per day increase in red/processed meat consumption, there was a 1.8% increased risk in cardiovascular mortality (HR: 1.018; 95% CI: 1.014, 1.022) (Figure 3B) with a significant increase in the risk of cardiovascular mortality (*P* < 0.0001) with higher meat intake. The nonlinear trend was not significant (*P* = 0.06) (Supplemental Table 7).

### Dairy products

**FIGURE 5:**
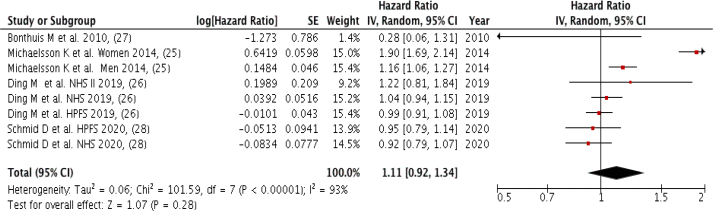
Forest plot showing multivariate adjusted HR with 95% CIs for the highest vs. the lowest dairy consumption and cardiovascular mortality in adults. 95% CI calculated from random effect models; IV, Inverse variation; pooled estimates of >1 favor higher consumption and of <1 favor lower consumption; NHS, Nurses’ Health Study; HPFS, Health Professional Health study.

Four studies (eight groups) with a total of 29,990 cardiovascular mortality cases were included in the meta-analysis by comparing the highest to the lowest dairy product intake, with no significant association observed (Pooled HR: 1.11; 95% CI: 0.92, 1.34; *P* = 0.28; I^2^ = 93%, *P*-heterogeneity < 0.00001) and very high between-study heterogeneity (Figure 5). There was no severe asymmetry observed from the visual inspection of the funnel plot (Supplemental Figure 3).

There was no significant association observed between dairy consumption and cardiovascular death risk (*P* = 0.13) in the dose-response analysis of four studies (eight groups) (Figure 3C). The nonlinear trend is not significant (*P* = 0.11) (Supplemental Table 7).

### Nuts

**FIGURE 6:**
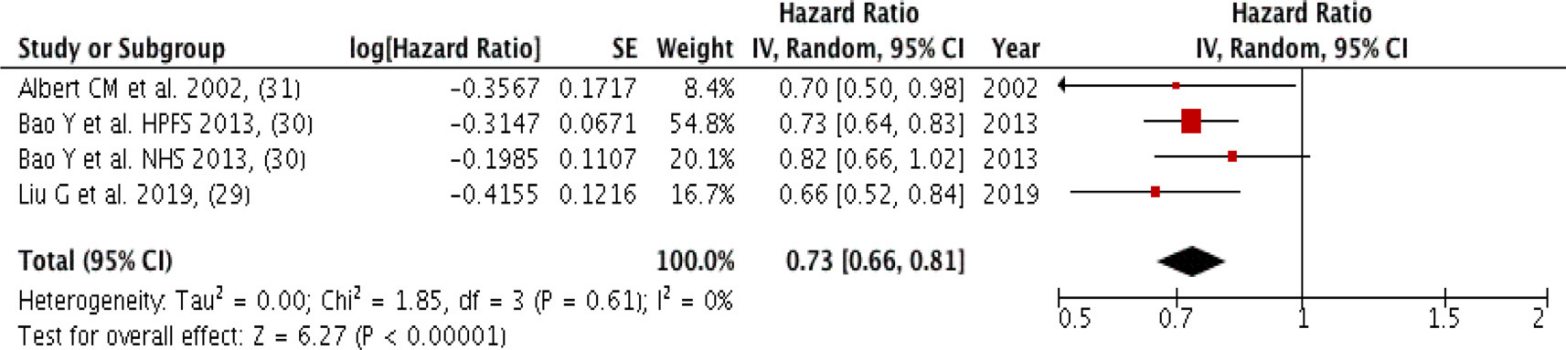
Forest plot showing of multivariate adjusted HR with 95% CIs for the highest vs. the lowest nut consumption and cardiovascular mortality for in adults. 95% CI calculated from random effect models; IV, Inverse variation; pooled estimates of >1 favor higher consumption and of <1 favor lower consumption; NHS, Nurses’ Health Study; HPFS, Health Professional Health study.

Three studies (four groups) with a total of 8700 cardiovascular mortality cases were included in the meta-analysis, comparing the highest intake to the lowest nut intake. A strong inverse association was observed (Pooled HR: 0.73; 95% CI: 0.66, 0.81; *P* < 0.00001; I^2^ = 0%, *P*-heterogeneity = 0.61), and zero heterogeneity among the included studies (Figure 6). There was no severe asymmetry observed from the visual inspection of the funnel plot (Supplemental Figure 4).

### Legume intake

**FIGURE 7:**
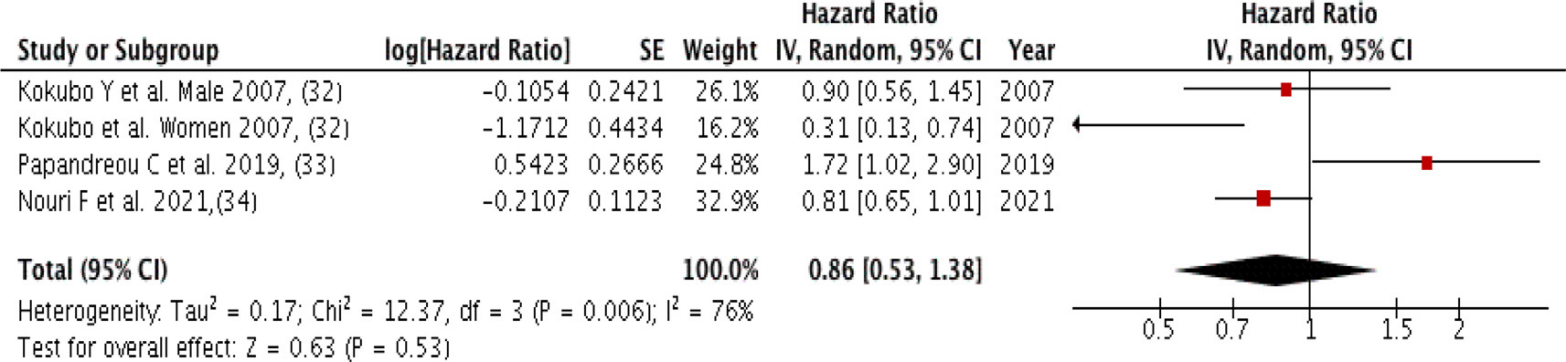
Forest plot showing multivariate adjusted HR with 95% CIs for the highest versus the lowest legume consumption and cardiovascular mortality in adults. 95% CI calculated from random effect models; IV, Inverse variation; pooled estimates of >1 favor higher consumption and of <1 favor lower consumption; NHS, Nurses’ Health Study; HPFS, Health Professional Health study.

Three studies (four groups) with a total of 1086 cardiovascular mortality cases were included in the meta-analysis, comparing the highest intake to the lowest legume intake, with no association observed (Pooled HR: 0.86; 95% CI: 0.53, 0.1.38; *P* = 0.53; I^2^ = 76%, *P*-heterogeneity = 0.006), and high heterogeneity among the included studies (Figure 7). There was no severe asymmetry observed from the visual inspection of the funnel plot (Supplemental Figure 5).

However, higher legume consumption was associated with a significant decrease in the risk of cardiovascular death (*P* = 0.02) in the dose-response analysis of three (four groups) included studies. The nonlinear trend was nonsignificant (*P* = 0.31). For each 10-gram per week increase in legume consumption, there is a 0.5% reduction in the risk of cardiovascular mortality (HR = 0.995, 95% CI: 0.991, 0.999) (Figure 3D).

### Fruits and vegetables

**FIGURE 8:**
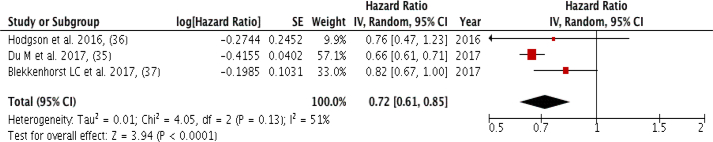
Forest plot showing multivariate adjusted HR with 95% CIs for the highest vs. the lowest fruits/vegetables consumption and cardiovascular mortality in adults. 95% CI calculated from random effect models; IV, Inverse variation; pooled estimates of >1 favor higher consumption and of <1 favor lower consumption; NHS, Nurses’ Health Study; HPFS, Health Professional Health study.

Three studies with a total of 6529 cardiovascular mortality cases were included in the highest intake quintile compared with the lowest intake quintile of fruits or vegetables meta-analysis. An inverse association (Pooled HR: 0.72; 95% CI: 0.61, 0.85; *P* < 0.0001; I^2^ = 51%, *P*-heterogeneity = 0.13) with low heterogeneity among the included studies was observed (Figure 8).

The meta-analysis showed that among the studies that examined fruit intake, the inverse association was greater (Pooled HR: 0.66; 95% CI: 0.61, 0.72; *P* < 0.00001; I^2^ = 0%, *P*-heterogeneity = 0.57) with zero heterogeneity among the studies (Supplemental Figure 6). None of the studies had reported an effect for vegetables intake only. There was no severe asymmetry observed from the visual inspection of the funnel plot (Supplemental Figure 7).

## Discussion

Our study analyzed the results from prospective cohort studies that investigated the association between the long-term intake of food groups and cardiovascular mortality. The findings indicate that the long-term consumption of fruits and vegetables, as well as whole grains and nuts, reduced the risk of cardiovascular mortality, whereas the long-term consumption of red/processed meat increased the risk of cardiovascular mortality in the meta- and dose-response analysis. Long-term consumption of dairy products had no effect on cardiovascular mortality in the meta- and dose-response analysis. Legume intake was not associated with cardiovascular mortality in the meta-analysis, but had an inverse association with cardiovascular mortality in the dose-response analysis.

We found that each 10-gram increase in whole grain intake per day is associated with a 4% reduction in the risk of cardiovascular mortality. Compared with the lowest intake of whole grain intake, people with the highest whole grain intake had a 13% lower risk of cardiovascular mortality. Similarly, our results showed a 27% lower risk of cardiovascular mortality in individuals with the highest nut intake compared with the lowest intake. Previous systematic reviews reported similar (18%–19%) reductions of risk of cardiovascular mortality with a higher whole grain intake [38,39]. Evidence from 16 countries [40], as well as a meta-analysis of five studies [41], reported the protective effects of nut consumption and cardiovascular mortality, which is consistent with our findings. In addition, our meta-analysis highlighted that long-term higher fruit and vegetable intake was associated with lower cardiovascular mortality by 28%, where a larger (36%) risk reduction was found with only higher fruit intake. These results are consistent with a review from 18 countries [42] that reported an inverse association between fruit and vegetable intake with cardiovascular mortality. A meta-analysis also reported a 4% risk reduction in cardiovascular mortality with each additional increase in serving of fruits and vegetables with no additional beneficial effects after consuming five servings of fruit and vegetables combined [43]. However, an umbrella review showed no significant association with cardiovascular mortality with higher fruit and vegetable intake [3]. These differences in risk might be due to methodological variation, in particular, the selection criteria of the included studies in the different reviews. By tracking long-term food group consumption, our study findings emphasized the protective effects of whole grains, fruits and vegetables, and nut intake on cardiovascular mortality.

Our study showed that each 10-gram increase in red/processed meat consumption per day is associated with a 1.8% increased risk of cardiovascular mortality, with individuals consuming the highest intakes having a 23% increased risk of cardiovascular mortality compared with those consuming the lowest amount. A previous meta-analysis by Abete et al. also reported harmful effects of red and processed meat intake on cardiovascular mortality, where red meat intake increased cardiovascular mortality risk by 16% and processed meat increased it by 18% [44]. Similarly, a review by Wang et al. [45] found an increased risk of cardiovascular mortality with processed meat intake among Asian and European populations. This harmful effect may be due to saturated and trans-fat contents in red and processed meat, which are associated with increased risk of hypercholesterolemia, endothelial dysfunction, insulin resistance, and type 2 diabetes that contribute to cardiovascular mortality [46]. Conversely, a meta-analysis conducted by Kim et al. [47] did not find an association between red meat consumption with stroke mortality. Further research is encouraged to confirm the effects of long-term red/processed meat consumption on stroke mortality.

Neither our meta- nor dose-response analysis showed effects of dairy intake on the risk of cardiovascular mortality. In line with our findings, a previous systematic review also reported no effects of dairy intake with the risk of coronary heart disease, regardless of the amount of dairy intake [48]. However, another systematic review reported that total dairy consumption lowered the risk of cardiovascular mortality (pooled effect size: 0.93), whereas high-fat milk consumption (highest versus lowest intake) was associated with a higher risk of cardiovascular mortality (pooled effect size: 1.917) [49]. The difference in findings could be explained through a single measurement of dairy intake in the included studies in previous reviews whereas our study considered the long-term consumption of dairy intake.

We found no associations of legume intake with the risk of cardiovascular mortality in our meta-analysis, which is supported by a previous systematic review by Li et al. [50]. However, in our dose-response analysis, we found that a 10-gram increase in legume intake per week is associated with a negligible 0.5% reduction in the risk of cardiovascular mortality. This discrepancy between the meta-analysis and dose-response analysis might be due to the exclusion of the Papandreou et al. [33] study as it had insufficient information to perform the dose-response analysis. The beneficial role of legumes in reducing cardiovascular mortality has been previously recognized as they contain high amounts of phytosterols that reduce serum total cholesterol and low-density lipoprotein cholesterol and increase the high-density lipoprotein cholesterol [51]. High levels of dietary fiber in legumes also associated with lower cardiovascular risk due to its low-density lipoprotein cholesterol-binding capability [52]. Consumption of legumes also tends to replace red meat consumption, which reduces saturated fat intake, further reducing cardiovascular risk [53].

### Strengths and limitations of the study

To the best of our knowledge, this is the first comprehensive review to evaluate the long-term effects of food group intake and cardiovascular mortality by only including studies that had repeated measures of dietary intake. In addition, we have only included prospective cohort studies in our meta-analysis and performed a dose-response analysis that provides robust information.

There is a need to interpret the results with some caution. First, all the included studies used self-reported measures to assess dietary intake, which may potentially introduce measurement bias. However, compared with other simple self-reported dietary measurements (e.g., only answering yes/no for specific food consumption), all studies included in our analysis used detailed food frequency questionnaires to measure dietary consumption that provided reasonable self-reported dietary data. Second, there were only few studies available based on our inclusion criteria, especially on nuts, fruits, and vegetables which limit our dose-response analysis. Third, although we attempted to explore sex-specific dietary habits related to cardiovascular mortality, there were very limited studies that allow us to do so. Fourth, although it would be interesting to examine long-term dietary exposures and specific types of cardiovascular mortality (such as stoke, myocardial infarction), there are limited studies available that allow us to perform further analysis. Fifth, though an exploratory study on the interactions between food groups in relation to cardiovascular mortality would have been desirable, the included studies did not provide sufficient information to perform further analysis. Last, the specificity of dietary exposure, such as the level of fat, and/or source (animal or plant derived) in dairy products were rarely mentioned in the included studies, which might lead to very high heterogeneity between studies.

In addition, there are some limitations due to the nature of observational studies that need to be acknowledged: [1] a causal association between CVD mortality and food intake cannot be determined from observational studies, [2] residual confounding in the original studies cannot be ruled out, and [3] the risk of making a type I error is increased due to conducting several statistical analyses without an alpha level adjustment.

## Conclusions

Although a major proportion of the studies evaluated dietary intake and cardiovascular mortality, the studies used one data point for dietary analyses, and thus limited studies have reviewed long-term dietary habits in relation to cardiovascular mortality. We included 22 longitudinal studies which emphasized the benefit of long-term high consumption of whole grains and nuts, as well as fruit and vegetables, and the harmful effects of long-term red/processed meat intake in relation to cardiovascular mortality. However, we were unable to draw a conclusion on the effects of specific food groups, such as fish and poultry on cardiovascular mortality, as there are limited studies that tracked long-term dietary habits. We encourage more data on legume intake and cardiovascular mortality.

## Funding

The study was supported by University of New South Wales [UNSW] School of Population Health Grant Building Support Scheme. XX is supported by a Post-doctoral Fellowship funded by the Heart Foundation of Australia (Award No. 102597) and Scientia Program at the UNSW, Australia.

## Author disclosures

Australia. All other authors report no conflicts of interest.

## Data availability

Data analyzed in this review were exacted from existing publications, which are openly available at locations cited in the reference section.
