# Supplementary material for: Long-Term Consumption of 10 Food Groups and Cardiovascular Mortality: A Systematic Review and Dose Response Meta-Analysis of Prospective Cohort Studies
Source: Adv Nutr. 2022 Dec 22;14(1):55–63. doi: 10.1016/j.advnut.2022.10.010 (PMC10102997; doi:10.1016/j.advnut.2022.10.010)
Supplement: Multimedia component 1 [file mmc1.docx]

**Supplementary figures**


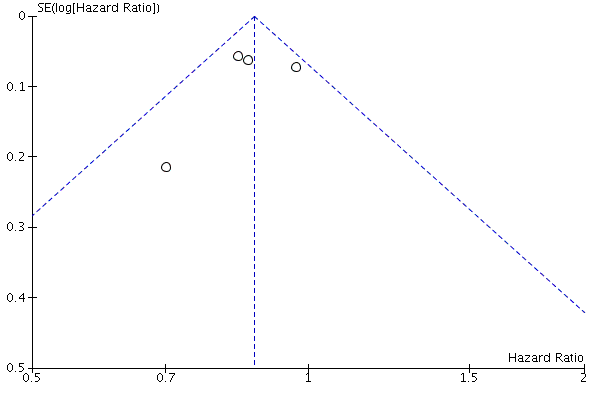


Supplementary Figure 1: Funnel plot showing study precision against the hazard ratio with 95% CIs for whole grains intake and cardiovascular mortality in adults


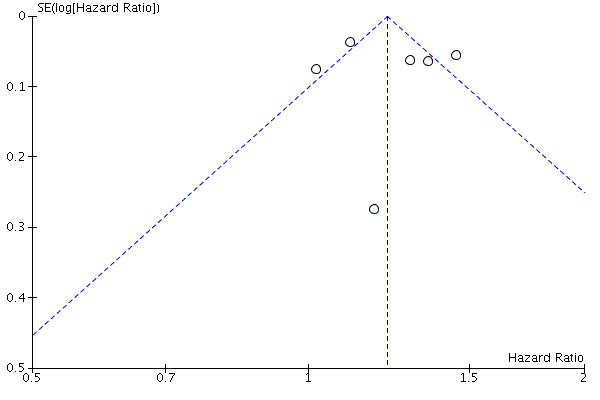


Supplementary Figure 2: Funnel plot showing study precision against the hazard ratio with 95% CIs for red and processed meat and cardiovascular mortality in adults


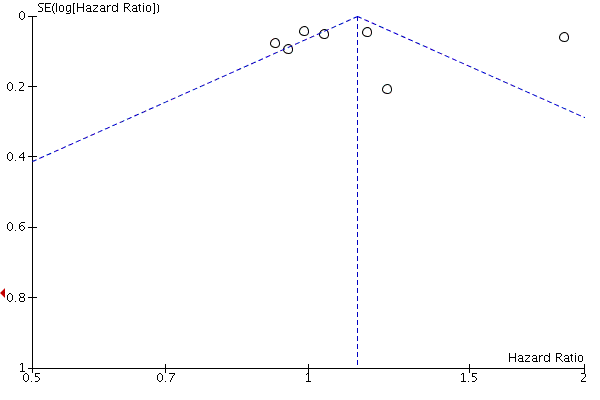


Supplementary Figure 3: Funnel plot showing study precision against the hazard ratio with 95% CIs for dairy and cardiovascular mortality in adults


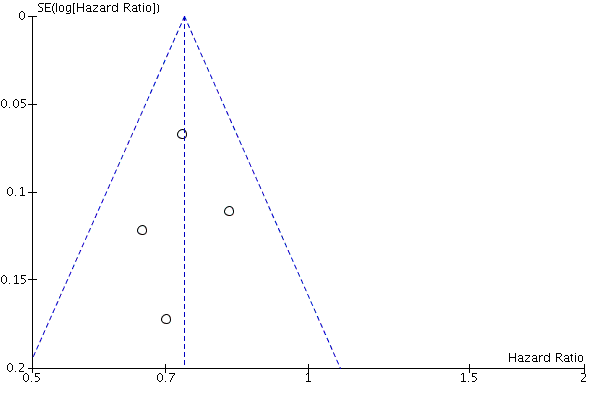


Supplemental Figure 4: Funnel plot showing study precision against the hazard ratio with 95% CIs for nut intake and cardiovascular mortality in adults


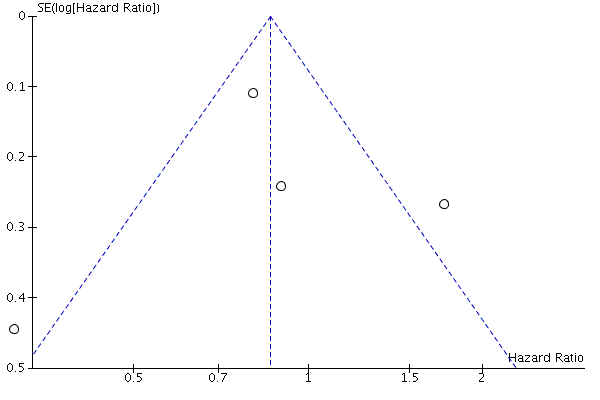


Supplemental Figure 5: Funnel plot showing study precision against the hazard ratio with 95% CIs for legume intake and cardiovascular mortality in adults


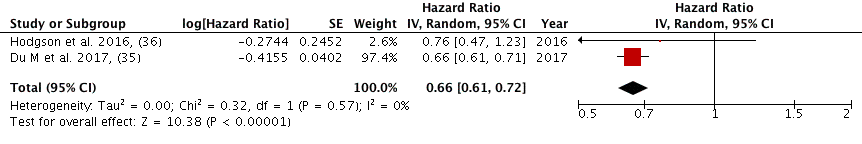


Supplementary Figure 6: Forest plot showing multivariate adjusted Hazard Ratio (HR) with 95% CI for highest versus lowest fruits intake and cardiovascular mortality in adults. 95% CI, 95% conﬁdence interval; calculated from random effect models (excluding vegetable studies)


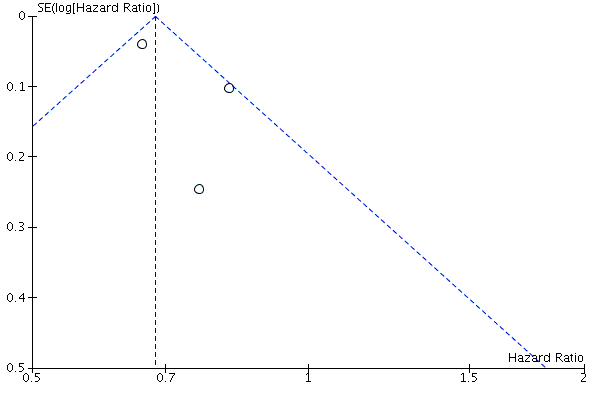


Supplemental Figure 7: Funnel plot showing study precision against the hazard ratio with 95% CIs for fruits and vegetable intake and cardiovascular mortality in adults
