# Supplementary material for: Long-Term Consumption of 10 Food Groups and Cardiovascular Mortality: A Systematic Review and Dose Response Meta-Analysis of Prospective Cohort Studies
Source: Adv Nutr. 2022 Dec 22;14(1):55–63. doi: 10.1016/j.advnut.2022.10.010 (PMC10102997; doi:10.1016/j.advnut.2022.10.010)
Supplement: Multimedia component 2 [file mmc2.docx]

**MEDLINE Search strategies**

| **Subjects** | **Keywords** | **Search strategy**  **Medline** |
| --- | --- | --- |
| Food group categories | **Vegetable**  **Fruit and** Fruit juice  **Grains**  **Dairy products**  **Meat & poultry, Fish, Eggs**  **Beans/legumes**  Nuts/seeds | **Vegetable**  “Vegetables”/or Vegetables.mp or Veg*.mp or Food/  **Fruit**  Fruit/or fruit.mp or Fruit*.mp or  Fruit and vegetable “juices”/ or fruit juice.mp  **Grains**  Whole grains/ or Edible grain/ or grains.mp or grains*.mp Or cereal*.mp. or refined grains.mp,  **Dairy Products**  Dairy Products/ or dairy products.mp or  Cheese/ or cheese.mp or  Yogurt/ or yogurt.mp or  Milk/ or milk.mp or milk*.mp or milk products  Ice cream  **Meat and poultry**  Meat/ or meat.mp or Meat Products/ or meat products.mp or Red meat/ or red meat.mp or lean meat.mp or processed meat.mp  Poultry/ or poultry products/ or poultry.mp or  Eggs/ or eggs.mp or eggs products.mp  Fishes/ or fish.mp or fish products/ fish products.mp, Seafood/ Or seafood.mp  **Legumes and Beans**  Legumes.mp or beans.mp or  Nuts/ or nuts.mp or  Seeds/ or Seeds.mp or almond.mp  Pulses.mp or Soy Foods/ or Tofu.mp or soybeans/ or soybean.mp |
| Cardiovascular disease mortality | Cardiovascular mortality  Major acute cardiovascular event | Death/ or Death.mp or Death, Sudden/ or Death, Sudden, Cardiac/OR mortality or cardiovascular mortality AND cardiovascular disease.mp or cardiovascular diseases/ or stroke/ or stroke.mp OR cerebrovascular disorders/ or cerebrovascular disease.mp OR cerebrovascular accident.mp OR cerebral infarction or cerebral haemorrhage/ or intracerebral haemorrhage.mp or subarachnoid haemorrhage/ or subarachnoid haemorrhage.mp or coronary disease/ or coronary heart disease.mp OR coronary artery disease/ or coronary artery diseae.mp OR myocardial Ischemia/ or acute coronary syndrome/ or angina pectoris/ or myocardial infraction/ or acute myocardial infarction.mp OR myocardial infarction Or acute coronary syndrome/ or acute coronary syndrome.mp OR heart failure/ or heart failure.mp OR cardiac failure.mp OR cardiac insufficiency.mp or cardiac arrest.mp or heart arrest/ or congestive heart failure.mp or heart attack.mp or Cardiovascular events.mp or Major acute cardiovascular event.mp. AND |
| Study design | Prospective, cohort, longitudinal | prospective studies/ or prospective.mp OR follow-up OR cohort analysis/ or cohort studies.mp OR longitudinal study/ or longitudinal.mp or case cohort or nested case control.mp |

**EMBASE search strategies**

| **Subjects** | **Keywords** | **Search strategy**  **Medline** |
| --- | --- | --- |
| Food group categories | **Vegetable**  Vegetables  **Fruit**  Fruit  Fruit juices  **Grains**  Whole grains, high cereal fibre  **Dairy**  Milk  Yogurt  Cheese  **Lean meat & poultry**  Fish, Eggs  **Beans/legumes**  Beans/legumes  Nuts/seeds | **Vegetable**  vegetables/ [MeSH] or Vegetables.mp or Veg*.mp or  **Fruit**  fruit or Fruit.mp or  Fruit*.mp or fruit vegetable/ OR fruit.mp. or fruit vegetable/ or fruit/ or Fruit and vegetable juices/ or fruit juice/ or fruit juice*.mp or  **Grains**  Whole grains/ or whole grains.mp. or grain/ or grain.mp or Food grain/ or Edible grain.mp or cereal/or breakfast cereal/ or processed cereal/ or cereal-based diet/ or cereal.mp or refined grains.mp. or refined grain/  **Dairy Products**  Dairy Product/ or dairy product.mp or  cheese/ or Cheese.mp or Yogurt.mp or yoghurt/ or  milk/ or Milk.mp or milk*.mp or milk products or ice cream.mp. or ice cream/  **Lean meat and poultry**  meat/ or meat.mp. or red meat/ or processed meat/ or poultry meat.mp or lean meat.mp or poultry meat/ or Poultry/ or poultry.mp or poultry product/ or poultry products.mp  egg/ or Eggs.mp or eggs products.mp  fish/ or Fish.mp or fish product/ fish product.mp or seafood  **Legumes and Beans**  Legumes.mp or legume/ or bean/ or bean.mp or Nuts.mp. or nut/ or Seed.mp. plant seed/ or almond.mp. or almond/ or Pulses.mp or Soy Foods/ or Soy Foods.mp orTofu.mp or soybeans/ or soybean.mp |
| Cardiovascular disease mortality | Cardiovascular mortality  Major acute cardiovascular event | death/ or sudden death/ or Death.mp. or sudden cardiac death/ OR mortality/ or cardiovascular mortality/ or mortality.mp **AND**  cardiovascular disease.mp or cardiovascular diseases/ or stroke.mp OR cerebrovascular accident/ OR cerebrovascular disorders .mp or cerebrovascular disease/ OR cerebral infarction.mp or brain infraction/ or cerebral haemorrhage/ or brain haemorrhage/ or intracerebral haemorrhage.mp or subarachnoid haemorrhage/ or subarachnoid haemorrhage.mp or coronary disease.mp or coronary artery disease/ OR myocardial ischemia.mp or heart muscle ischemia/ or angina pectoris/ or myocardial infraction.mp or heart infraction/ or acute coronary syndrome.mp or acute coronary syndrome acute heart infraction/ or unstable angina pectoris/ OR heart failure/ or heart failure.mp OR cardiac failure.mp OR cardiac insufficiency.mp or cardiac arrest.mp or heart arrest/ or congestive heart failure.mp or congestive heart failure/ or heart attack.mp or Cardiovascular events.mp or Major acute cardiovascular event.mp |
| Study design | Prospective, cohort, longitudinal | prospective studies/ or prospective.mp OR follow-up OR cohort anlaysis/ or cohort studies.mp OR longitudinal study/ or longitudinal.mp or case cohort or nested case control.mp |

**CINAHL search strategies**

Vegetable* OR Veg* OR Fruit*OR "Fruit and vegetable*" OR "Fruit and vegetable juice*" OR "Fruit juice*" OR grain* OR "Whole Grain*" OR "Edible Grain*" OR Cereal* OR "refined grains*" OR "Dairy Product*" OR dairy* OR cheese* OR "Cheese product*" OR yogurt* OR milk* OR "milk product*" OR "Ice cream*” OR Meat* OR "Meat Product*" OR "Red Meat *" OR "lean meat *" OR "Processed meat*" OR poultry* OR "poultry product*" OR egg* OR "eggs product*" OR fish* OR "fish product*" OR seafood* OR legume* OR bean* OR nut* OR Seed* OR Pulses* OR "Soy Foods*" OR Tofu* OR Soybean*

**AND**

“Death* OR “Death, Sudden*” OR “Death, Sudden, Cardiac*” OR mortality*OR “cardiovascular mortality*”

**AND**

“cardiovascular disease*” OR stroke* OR “cerebrovascular disorders*” OR “cerebrovascular disease*” OR “cerebrovascular accident*” OR “cerebral infarction*” OR “cerebral haemorrhage*” OR “intracerebral haemorrhage*” OR “subarachnoid haemorrhage*” OR “coronary disease*” or “coronary heart disease*” OR “coronary artery disease*” OR “myocardial Ischemia*” or “acute coronary syndrome*” or “angina pectoris*” or myocardial infraction*” or “acute myocardial infarction*” OR “heart failure*” OR “cardiac failure*” OR “cardiac insufficiency*” or “cardiac arrest*” or “heart arrest*” or “congestive heart failure*” or “heart attack*” or “Cardiovascular events*” or “Major acute cardiovascular event*”

**AND**

“prospective studies*” OR “follow up*” OR “cohort analysis*” or “cohort studies*”OR “longitudinal study*” or longitudinal* or “case cohort*” or “nested case-control*”

**Web of Science search strategies**

Vegetable* OR Veg* OR Fruit*OR "Fruit and vegetable*" OR "Fruit and vegetable juice*" OR "Fruit juice*" OR grain* OR "Whole Grain*" OR "Edible Grain*" OR Cereal* OR "refined grains*" OR "Dairy Product*" OR dairy* OR cheese* OR "Cheese product*" OR yogurt* OR milk* OR "milk product*" OR "Ice cream*” OR Meat* OR "Meat Product*" OR "Red Meat *" OR "lean meat *" OR "Processed meat*" OR poultry* OR "poultry product*" OR egg* OR "eggs product*" OR fish* OR "fish product*" OR seafood* OR legume* OR bean* OR nut* OR Seed* OR Pulses* OR "Soy Foods*" OR Tofu* OR Soybean*

**AND**

“Death* OR “Death, Sudden*” OR “Death, Sudden, Cardiac*” OR mortality*OR “cardiovascular mortality*”

**AND**

“cardiovascular disease*” OR stroke* OR “cerebrovascular disorders*” OR “cerebrovascular disease*” OR “cerebrovascular accident*” OR “cerebral infarction*” OR “cerebral haemorrhage*” OR “intracerebral haemorrhage*” OR “subarachnoid haemorrhage*” OR “coronary disease*” or “coronary heart disease*” OR “coronary artery disease*” OR “myocardial Ischemia*” or “acute coronary syndrome*” or “angina pectoris*” or myocardial infraction*” or “acute myocardial infarction*” OR “heart failure*” OR “cardiac failure*” OR “cardiac insufficiency*” or “cardiac arrest*” or “heart arrest*” or “congestive heart failure*” or “heart attack*” or “Cardiovascular events*” or “Major acute cardiovascular event*”

**AND**

“prospective studies*” OR “follow up*” OR “cohort analysis*” or “cohort studies*”OR “longitudinal study*” or longitudinal* or “case cohort*” or “nested case-control*”

**SCOPUS search strategies**

Vegetable* OR Veg* OR Fruit*OR "Fruit and vegetable*" OR "Fruit and vegetable juice*" OR "Fruit juice*" OR grain* OR "Whole Grain*" OR "Edible Grain*" OR Cereal* OR "refined grains*" OR "Dairy Product*" OR dairy* OR cheese* OR "Cheese product*" OR yogurt* OR milk* OR "milk product*" OR "Ice cream*” OR Meat* OR "Meat Product*" OR "Red Meat *" OR "lean meat *" OR "Processed meat*" OR poultry* OR "poultry product*" OR egg* OR "eggs product*" OR fish* OR "fish product*" OR seafood* OR legume* OR bean* OR nut* OR Seed* OR Pulses* OR "Soy Foods*" OR Tofu* OR Soybean*

**AND**

“Death* OR “Death, Sudden*” OR “Death, Sudden, Cardiac*” OR mortality*OR “cardiovascular mortality*”

**AND**

“cardiovascular disease*” OR stroke* OR “cerebrovascular disorders*” OR “cerebrovascular disease*” OR “cerebrovascular accident*” OR “cerebral infarction*” OR “cerebral haemorrhage*” OR “intracerebral haemorrhage*” OR “subarachnoid haemorrhage*” OR “coronary disease*” or “coronary heart disease*” OR “coronary artery disease*” OR “myocardial Ischemia*” or “acute coronary syndrome*” or “angina pectoris*” or myocardial infraction*” or “acute myocardial infarction*” OR “heart failure*” OR “cardiac failure*” OR “cardiac insufficiency*” or “cardiac arrest*” or “heart arrest*” or “congestive heart failure*” or “heart attack*” or “Cardiovascular events*” or “Major acute cardiovascular event*”

**AND**

“prospective studies*” OR “follow up*” OR “cohort analysis*” or “cohort studies*” OR “longitudinal study*” or longitudinal* or “case cohort*” or “nested case-control*”
