# Supplementary material for: Long-Term Consumption of 10 Food Groups and Cardiovascular Mortality: A Systematic Review and Dose Response Meta-Analysis of Prospective Cohort Studies
Source: Adv Nutr. 2022 Dec 22;14(1):55–63. doi: 10.1016/j.advnut.2022.10.010 (PMC10102997; doi:10.1016/j.advnut.2022.10.010)
Supplement: Multimedia component 3 [file mmc3.docx]

**Supplementary Table 1. Included study characteristics of investigating the association between whole grain intake and risk of cardiovascular mortality in adults**^1^

| **Author** | **Year** | **Country** | **Cohort name** | **Age at entry** | **Sex** | **Sample size** | **Total cases** | **Dietary assessment** | **Outcome assessment** | **Type of whole grains** | **Adjusted factors** | **Follow up years** | **Results**  **(The highest vs. the lowest intake category)**  **Hazard Ratio (95% CI)** | **New castle Ottawa tool score** |
| --- | --- | --- | --- | --- | --- | --- | --- | --- | --- | --- | --- | --- | --- | --- |
| He M et al. (17) | 2010 | USA | NHS | 30-55 | Female with Type 2 Diabetes Mellitus | 7,822 | 295 | SFFQ | National Registry | Whole grains | Age, smoking status, BMI, alcohol intake, physical activity, medical insurance, physical activity, parental history of MI, menopausal status and use of hormone therapy, and duration of diabetes mellitus, total energy, intakes of polyunsaturated, saturated, trans fat, magnesium and folate (all in quintiles). | 26 | 0.70 (0.46,1.06)  Quintile 5 Vs Quintile 1 | 8 |
| Eshak ES et al. (16) | 2014 | Japan | JPHC | 40-69 | Both | 92,223 | 2705 | SFFQ | National Registry | Rice | Age, sex, public health center area, history of hypertension, history of diabetes, use of lipid-lowering drugs, BMI, smoking status, ethanol intake, leisure-time sports activity, occupation, intakes of seafood, meat, fruit, vegetables, soy, SFAs, and sodium, total energy intake, and, for women, menopausal status and hormone use | 15-18 | 0.97 (0.84, 1.13)  251 ± 83 g/d  Vs  542 ± 127 g/d | 8 |
| Wu H et al. (18) | 2015 | USA | NHS | 30-55 | Women | 74341 | 2,989 | SFFQ | National Registry | Whole grains | Age, ethnicity, BMI, smoking, alcohol, physical activity, family history of diabetes, cancer and heart disease, multivitamin use, aspirin use at least once per week, history of hypertension, high cholesterol, or diabetes at baseline, energy intake, modified alternative healthy eating index, which did not include whole grains, postmenopausal status, postmenopausal hormone use | 26 | 0.86 (0.76, 0.96)  Quintile 5 Vs Quintile 1  4.3 g/d Vs 33 g/d | 8 |
| Wu H et al. (18) | 2015 | USA | HPHS | 32-87 | Men | 43,744 | 3,621 | SFFQ | National Registry | Whole grains | Age, ethnicity, smoking, alcohol intake, physical activity, family history of diabetes, cancer and heart disease, multivitamin use, aspirin use at least once per week, history of hypertension, high cholesterol, or diabetes at baseline, energy intake, modified alternative healthy eating index, which did not include whole grains | 24 | 0.84 (0.75, 0.93)  Quintile 5 Vs Quintile 1  5.9 g/d Vs 47.8 g/d | 8 |

^1^ BMI, Body Mass Index, CI, Confidence Interval; HPHS, Health Professionals Follow-Up Study; JPHC, The Japan Public Health Center–Based study; SFAs, Saturated Fatty Acids; SFFQ, Self-administered Food Frequency Questionnaire; MI, Myocardial Infraction; NHS, The Nurses’ Health Study; USA, United States

**Supplementary Table 2. Included study characteristics of investigating the association between red Meat intake and risk of cardiovascular mortality in adults^1^**

| **Author** | **Year** | **Country** | **Cohort name** | **Age at entry** | **Sex** | **Sample size** | **Total cases** | **Dietary assessment** | **Outcome assessment** | **Type of meat** | **Adjustment factors** | **Follow-up years** | **Results**  **(The Highest vs. the lowest intake category)**  **Hazard Ratio (95% CI)** | **New castle Ottawa tool score** |
| --- | --- | --- | --- | --- | --- | --- | --- | --- | --- | --- | --- | --- | --- | --- |
| Bellavia A et al. (19) | 2016 | Sweden | COSM and SMC | 48-83- F  45-79- M | Both | 74,645 | 5,495 | SFFQ | National death register | Red meat | Sex, smoking, physical activity, education, BMI, alcohol, diabetes, fish, and energy intake | 16 | 1.29 (1.14, 1.46)  140 vs. 31 g/d (median) | 8 |
| Pan A et al. (20) | 2012 | USA | HPFS | 40-75 | Men | 37,698 | 2,716 | SFFQ | National Registry | Red meat | Age, BMI, alcohol, physical activity, smoking, race, family history of diabetes, myocardial infarction, cancer; history of diabetes, hypertension, or hypercholesterolemia; energy intake, whole grains, fruits, and vegetables | 22 | 1.35 (1.19, 1.53)  Quintile 5 Vs Quintile 1  2.07 vs. 0.25 servings/d | 8 |
| Pan A et al. (20) | 2012 | USA | NHS | 30-55 | Women | 83,644 | 3,194 | SFFQ | National Registry | Red meat | Age, BMI, alcohol, physical activity, smoking, race, family history of diabetes, myocardial infarction, cancer; history of diabetes, hypertension, or hypercholesterolemia, energy intake, whole grains, fruits, and vegetables | 28 | 1.45 (1.30, 1.63)  Quintile 5 Vs Quintile 1  2.17 vs. 0.51 servings/d | 8 |
| Zheng Y et al. (22) | 2013 | USA | NHS  HPFS | 40-70 -M  30-55-F | Both | 81,469 | 3,528 | SFFQ | National Registry | Red meat | Age, energy intake, income, occupation, education, comorbidity index, physical activity level, vegetable, fruit, fish, and red meat or poultry, smoking, alcohol | 12 | 1.11(1.03, 1.20)  Increase of 1g per serving | 6 |
| McCullough ML. et al. (21) | 2013 | USA | Cancer prevention study II  Nutrition cohort | 40-93 cancer survivors | Both | 2,315 | 176 | SFFQ^2^ | National Registry | Red and processed meat | age at diagnosis, sex, tumor stage at diagnosis, prediagnostic energy intake, and postdiagnostic energy intake (both sex-specific quartiles) | 17 | 1.18 (0.69, 2.04)  10.4 mean serving/wk Vs  1.5 mean serving/wk  Low pre/post dignosis Vs High pre/post dignosis | 6 |
| Appleby PN et a. (23) | 2016 | United Kingdom | OVS and the EPIC Oxford cohort | 20-89 | Both | 78,741 | 542 | SFFQ | Death certificates | Meat | Smoking, alcohol consumption, physical activity, whether married or cohabiting and regular use of nutritional supplements and stratified by study/method of recruitment; all possible combinations of sex, parity, oral contraceptive use, and hormone therapy use, prior diabetes, prior high blood pressure, and receipt of long-term medical treatment, with the use of separate models for each endpoint, BMI | 9 | 0.98 (0.85, 1.13)  1.02(0.88, 1.17)inverse  Low meat eater’s vs regular meat eaters  Less frequent Vs ≤ 5 times/wk | 7 |
| Nakamura Y et al. (24) | 2004 | Japan | National Survey on Circulatory Disorders  (NIPPON DATA80) | ≥30 | Both | 9263 | 1202 | SFFQ^2^ | National Registry | Eggs | Age, serum creatinine, cholesterol, blood glucose, BMI, systolic and diastolic blood pressure, drugs, smoking, alcohol | 14 | Stroke  M= 0.25 (0.03, 1.81)  F = 1.22 (0.29, 5.17)  IHD  F= 1.27 (0.16, 9.80  >2 vs 1 servings/d | 8 |

^1^ BMI, Body Mass Index, CI, Confidence Interval; COSM, Cohort of Swedish Men; F, Female; HPHS, Health Professionals Follow-Up Study; IHD, Ischemic Heart Disease; JPHC, The Japan Public Health Center–Based study; SFFQ, Self-administered Food Frequency Questionnaire; SMC, The Swedish Mammography Cohort; M, Male; MI, Myocardial Infraction; NHS, The Nurses’ Health Study; OVS, Oxford Vegetarian Study; USA, United States

^2.^ used brief blocked FFQ, which is also a self-administered FFQ (Block G, Hartman AM, Naughton D. A reduced dietary questionnaire: development and validation. *Epidemiol* 1990 Jan;1(1):58-64.)

**Supplementary Table 3. Included study characteristics of investigating the association between dairy products and risk of cardiovascular mortality in adults^1^**

| **Author** | **Year** | **Country** | **Cohort name** | **Age at entry** | **Sex** | **Sample size** | **Total cases** | **Dietary assessment** | **Outcome assessment** | **Type of dairy** | **Adjustment factors** | **Follow-up years** | **Results**  **(The highest vs. the lowest intake category)**  **Hazard Ratio (95% CI)** | **New castle Ottawa tool score** |
| --- | --- | --- | --- | --- | --- | --- | --- | --- | --- | --- | --- | --- | --- | --- |
| Bonthuis et al. (27) | 2010 | Australia | Residents of Nambour-  a skin cancer prevention trial | 25-78 | Both | 1,529 | 61 | SFFQ | National Registry | Dairy products | Age, Sex, energy intake, smoking, physical activity, BMI, alcohol, education, dietary supplement, b-carotene treatment, medical condition, calcium | 14.4 | 0.28 (0.06, 1.34)  599 vs. 174 g/d | 7 |
| Michaëlsson K et al. (25) | 2014 | Sweden | SMC | 39-74 | Women | 61,433 | 5,278 | SFFQ | National Registry | Milk | Age, energy intake, height, alcohol, dietary pattern, calcium, vitamin D, cortisone, living alone, education, BMI, smoking, physical activity, Charlson comorbidity index, oestrogen use, null parity | 20.1 | 1.90 (1.69, 2.14)  ≥3 glasses (≥600 g/d)  Vs < 1 glasses (< 200 g/d) | 7 |
| Michaëlsson K et al. (25) | 2014 | Sweden | COSM | 45-79 | Men | 45,339 | 4,568 | SFFQ | National Registry | Milk | Age, energy intake, height, alcohol, dietary pattern, calcium, vitamin D, cortisone, living alone, education, BMI, smoking, physical activity, Charlsons comorbidity index | 20.1 | 1.16 (1.06, 1.27)  ≥3 glasses (≥600 g/d)  Vs  < 1 glasses  (< 200 g/d) | 7 |
| Ding M et al. (26) | 2019 | USA | NHS | 30-55 | Women | 74,805 | 4,418 | SFFQ | National death Index | Dairy | Age, education, income, smoking, alcohol, multivitamin, menopausal status and hormone therapy, physical activity, BMI, waist-to-hip ratio, history of cardiovascular disease, diabetes, or hypertension, energy intake | 32 | 1.04 (0.94, 1.16)  Quintile 5 (4.09±1.15 servings/day)  Vs  Quintile 1 (0.67±0.28 servings/da) | 8 |
| Ding M et al. (26) | 2019 | USA | NHS II | 25-42 | Women | 93,348 | 258 | SFFQ | National death Index | Dairy | Age, education, income, smoking, alcohol, multivitamin, menopausal status and hormone therapy, physical activity, BMI, waist-to-hip ratio, history of cardiovascular disease, diabetes, or hypertension, energy intake | 27 | 1.22 (0.81, 1.83)  Quintile 5 (0.96 ±0.37 servings/day) Vs  Quintile 1  (6.43 ±2.52 servings/day) | 8 |
| Ding M et al. (26) | 2019 | USA | HPFS | 40-75 | Men | 49,602 | 7,467 | SFFQ^1^ | National death Index | Dairy | Age, education, income, smoking, alcohol, multivitamin, physical activity, BMI, waist-to-hip ratio, history of cardiovascular disease, diabetes, or hypertension, energy intake | 30 | 0.99 (0.91, 1.08)  (0.52 ±0.24 servings/day) Vs  4.00 ±1.29 servings/day) | 8 |
| Schmid D et al. (28) | 2020 | USA | NHS | 30-55 | Women | 82,348 | 4,207 | SFFQ | National Registry | Yogurt | Age, height, current BMI 18 yrs, smoking, alcohol, multivitamin, menopausal status and hormone therapy, physical activity, BMI, history of hypercholesterolemi, cancer, MI diabetes, or hypertension, intakes of unprocessed red meat, processed meat, nuts, total fiber, fruits, vegetables, and total calciumenergy intake | 32 | 0.92 (0.79, 1.08)  >4times/wk  Vs Never | 8 |
| Schmid D et al. (28) | 2020 | USA | HPFS | 40-75 | Men | 40,278 | 3,733 | SFFQ | National Registry | Yogurt | Age, height, current BMI 21 yrs, smoking, alcohol, multivitamin, physical activity, BMI, history of hypercholesterolemi, cancer, MI diabetes, or hypertension, intakes of unprocessed red meat, processed meat, nuts, total fiber, fruits, vegetables, and total calciumenergy intake | 26 | 0.95 (0.79, 1.13)  >4times/wk  Vs  Never | 8 |

^1^ BMI, Body Mass Index, CI, Confidence Interval; COSM, Cohort of Swedish Men; HPHS, Health Professionals Follow-Up Study; SFFQ, Self-administered Food Frequency Questionnaire; SMC, The Swedish Mammography Cohort; MI, Myocardial Infraction; NHS, The Nurses’ Health Study; USA, United States

**Supplementary Table 4. Included study characteristics of investigating the association between Nut consumption and risk of cardiovascular mortality in adults^1^**

| **Author** | **Year** | **Country** | **Cohort name** | **Age at entry** | **Sex** | **Sample size** | **Total cases** | **Dietary assessment** | **Outcome assessment** | **Type of nuts** | **Adjustment factors** | **Follow-up years** | **Results**  **(The highest vs. the lowest intake category)**  **Hazard Ratio (95% CI)** | **Quality** |
| --- | --- | --- | --- | --- | --- | --- | --- | --- | --- | --- | --- | --- | --- | --- |
| Bao Y et al. (30) | 2013 | USA | NHS | 30-55 | Women | 76,464 | 3,086 | SFFQ | National Registry | Nuts | Age, race, BMI, physical activity, smoking, multivitamin use, aspirin use, family history of diabetes mellitus, myocardial infarction, or cancer; history of diabetes mellitus, hypertension, or hypercholesterolemia, energy intake, alcohol, red or processed meat, fruits, vegetables, menopausal status and hormone use | 30 | 0.82 (0.66, 1.01)  ≥5servings/wk Vs  Never | 8 |
| Bao Y et al. (30) | 2013 | USA | HPFS | 32-87 | Men | 42,498 | 3,385 | SFFQ | National Registry | Nuts | Age, race, BMI, physical activity, smoking, multivitamin use, aspirin use, family history of diabetes mellitus, myocardial infarction, or cancer; history of diabetes mellitus, hypertension, or hypercholesterolemia, energy intake, alcohol, red or processed meat, fruits, and vegetables | 24 | 0.73 (0.64, 0.83)  ≥5 servings/wk Vs Never | 8 |
| Liu G et al. (29) | 2019 | USA | NHS  HPFS | 30-55-F  40-75-M | Both | 16,217 | 1,663 | SFFQ | National Registry | Nut | age ,diabetes mellitus duration (years), sex, race, BMI at diabetes mellitus diagnosis, smoking status, alcohol consumption, family history of MI or cancer , current aspirin use, hypertension, use of lipid-lowering medication, diabetes medication use, intake of total energy, red or processed meat, fruits, and vegetables | 34-NHS  HPFS- 28 yrs | 0.66 (0.52, 0.84)  ≥5 servings/wk VS <1 serving/mo | 8 |
| Albert CM et al. (31) | 2002 | USA | US Physicians' Health Study. | 40-84 | Male | 21,454 | 566 | SFFQ | National Registry | Nuts | Age, aspirin, beta carotene treatment, evidence of CVD before 12 months, BMI, smoking, DM, HTN, hypercholesterolemia, alcohol , vigorous exercise, vitamin C, E and multivitamin use at baseline, fish, meat consumption, fruit and veg and dairy intake at 12 month of follow up | 17 | 0.70 (0.50, 0.98)  >2serving/wk. Vs <1/mo | 8 |

^1^ BMI, Body Mass Index, CVD, Cardiovascular Disease; CI, Confidence Interval; DM, Diabetes; F, Female; HPHS, Health Professionals Follow-Up Study; SFFQ, Self-administered Food Frequency Questionnaire; M, Male; MI, Myocardial Infraction; NHS, The Nurses’ Health Study; WK, Week; USA, United States

**Supplementary Table 5. Included study characteristics of investigating the association between legumes and risk of cardiovascular mortality in adults^1^**

| **Author** | **Year** | **Country** | **Cohort name** | **Age at entry** | **Sex** | **Sample size** | **Total cases** | **Dietary assessment** | **Outcome assessment** | **Type of legumes** | **Adjustment factors** | **Follow-up years** | **Results**  **(The highest vs. the lowest intake category)**  **Hazard Ratio (95% CI)** | **Quality** |
| --- | --- | --- | --- | --- | --- | --- | --- | --- | --- | --- | --- | --- | --- | --- |
| Nouri F et al. (34) | 2021 | Iran | Isfahan cohort study | adults (≥35 years), | Both | 5,432 | 346 -F  405 -M | SFFQ | medical records, registries, hospital records, death certificates and  verbal autopsies | Legumes | Sex, age and time-varying confounders  including education, marital status, smoking, legume-adjusted dietary score, leisure-time  physical activity, BMI, anti-hyperlipidemic medicine, antihypertensive medicines and antidiabetic  medicine | 13 | 0.805 (0.650, 0.998)  1-3 times/ week intake Vs >3 intake/week | **7** |
| Papandreou C et al. (33) | 2019 | Spain | PREDIMED study | M= 55-80  F= 60-80 | Both | 7,212 | 103 | SFFQ | National Registry | Legumes | Age, sex and intervention group, prevalence of diabetes, prevalence of hypertension, hypercholesterolemia, baseline BMI, smoking, educational, physical activity, use of antihypertensive medication, antidiabetic agents, statin use, cumulative average of alcohol intake and cumulative average of the 13-point screener of Mediterranean diet adherence, total energy intake | 6 | 1.72 (1.02, 2.89)  Tertile 3 (27.34 g/day)  Vs  Tertile 1 (13.95 g/day) | 7 |
| Kokubo Y et al. (32) | 2007 | Japan | JPHC Study Cohort I | ≥35 | Women | 27,435 | 57 | SFFQ | National registry | Soy products | Age, sex, smoking, alcohol use, body mass index, history of hypertension or diabetes mellitus,medication use for hypercholesterolemia, education level, sports, dietary intake of fruits, vegetables, fish, salt, and energy, menopausal status for women | 12.5 | 0.31 (0.13, 0.74)  Group 3(≥ 5 times /wk)Vs Group 1 (0-2times/wk) | 7 |
| Kokubo Y et al. (32) | 2007 | Japan | JPHC Study Cohort I | 40-79 | Men | 27,063 | 175 | SFFQ | National registry | Soy products | Age,sex, smoking, alcohol use, body mass index, history of hypertension or diabetes mellitus, medication use for hypercholesterolemia, education level, sports, dietary intake of fruits, vegetables, fish, salt, and energy | 12.5 | 0.90 (0.56, 1.45)  Group 3 (≥ 5times /wk) Vs Group 1 (0-2times/wk) | 7 |

^1^ BMI, Body Mass Index, CI, Confidence Interval; F, Female; IHD, Ischemic Heart Disease; JPHC, The Japan Public Health Center–Based study; SFFQ, Self-administered Food Frequency Questionnaire; M, Male; MI, Myocardial Infraction; PREDIMED study, The PREvencion con DIeta MEDiterranea study; Wk, Week

**Supplementary Table 6. Included study characteristics of investigating the association between fruits and vegetables and risk of cardiovascular mortality in adults ^1^**

| **Author** | **Year** | **Country** | **Cohort name** | **Age at entry** | **Sex** | **Sample size** | **Total cases** | **Dietary assessment** | **Outcome assessment** | **Type of food** | **Adjusted factors** | **Follow-up years** | **Results**  **(The highest vs. the lowest intake category)**  **Hazard Ratio (95% CI)** | **Quality** |
| --- | --- | --- | --- | --- | --- | --- | --- | --- | --- | --- | --- | --- | --- | --- |
| Blekkenhorst LC et al. (37) | 2017 | Australia | Calcium Intake Fracture Outcome Study | ≥70 | Women | 1,226 | 128 | SFFQ | Death certificate | Total vegetables | age, body mass index, physical activity, alcohol intake, smoking history, socioeconomic status, calcium supplementation group, antihypertensive medication, statin medication, low-dose aspirin, Chronic Kidney Disease Epidemiology Collaboration estimated glomerular filtration rate and energy intake | 15 | 0.82 (0.67,1.00)  IHD  per 75 g/d for total vegetables | 7 |
| Du H et al. (35) | 2017 | China | China Kadoorie Biobank Study | 30-79 | Both | 462,342 | 6166 | Interviewer administered questionnaire | National registry | Fruits consumption | age, sex, region, smoking, alcohol intake, education, income, consumption of meat, dairy products and preserved vegetables, survey season, physical activity and BMI.  † Includes deaths with known causes other than infectious diseases, CVD, cancer and respiratory diseases | 7 | 0.66 (0.61, 0.71)  Regularly 4 days (1serving/day)/week Vs never/rarely | 8 |
| Hodgson et al. (36) | 2016 | Australia | The Calcium Intake Fracture Outcome Study | ≥70 | Women | 1,456 | 235 | SFFQ | Death certificate | Fruits | Age, BMI, treatment codes, smoking, socioeconomic status, history of diabetes, CVD, and cancer, hypertensive medications, cholesterol lowering medications, low-dose aspirin, physical activity, energy intake and alcohol intake | 15 | 0.76 (0.47, 1.24)  >100g/d Vs <5g/d | 7 |

^1^ BMI, Body Mass Index, CVD, Cardiovascular Disease; CI, Confidence Interval; IHD, Ischemic Heart Disease; SFFQ, Self-administered Food Frequency Questionnaire

**Supplementary Table 7: Summary statistics of the dose response analysis of the red/ processed meat, grains, dairy and legumes products in adults** ^1^

|  | Coefficient (se) | Pooled RR (10 unit) | 95% CI | p-value | I^2^ | P  Heterogeneity | P value for the Non-linear trends |
| --- | --- | --- | --- | --- | --- | --- | --- |
| Red/processed meat | 0.0018 (0.0002) | 1.018 | 1.014, 1.022 | <0.0001 | 0 | 0.56 | 0.06 |
| Grains | -0.004 (0.0008) | 0.96 | 0.95, 0.98 | <0.0001 | 0 | 0.50 | 0.23 |
| Dairy | 0.0003 (0.0002) | 1.003 | 0.999, 1.006 | 0.13 | 96.7% | <0.0001 | 0.11 |
| Legumes | -0.0005 | 0.995 | 0.991, 0.999 | 0.02 | 45.8% | 0.16 | 0.31 |

^1^ RR, Relative risk; CI, Confidence Interval
